# Treatment of acute sciatica with transforaminal epidural corticosteroids and local anesthetic: design of a randomized controlled trial

**DOI:** 10.1186/s12891-017-1571-8

**Published:** 2017-05-25

**Authors:** Bastiaan C. ter Meulen, Esther T. Maas, Amrita Vyas, Marinus van der Vegt, Koo de Priester, Michiel R. de Boer, Maurits W. van Tulder, Henry C. Weinstein, Raymond W. J. G. Ostelo

**Affiliations:** 1 Department of Neurology, Zaans Medisch Centrum, Zaandam and Onze Lieve Vrouwe Gasthuis, Amsterdam, The Netherlands; 2 Department of Health Sciences and the EMGO Institute for Health and Care Research, Faculty of Earth and Life Sciences, VU University Amsterdam, Amsterdam, The Netherlands; 3 Department of Neurology, Onze Lieve Vrouwe Gasthuis, Amsterdam, The Netherlands; 4 Department of Anesthesiology Zaans Medisch Centrum, Zaandam, The Netherlands; 5 Department of Radiology Zaans Medisch Centrum, Zaandam, The Netherlands; 6 Department of Epidemiology and Biostatistics, EMGO Institute for Health and Care Research, VU University Medical Centre Amsterdam, Amsterdam, The Netherlands

**Keywords:** Sciatica, Lumbar disc herniation, Transforaminal epidural steroids, Economic evaluation, Randomized controlled trial

## Abstract

**Background:**

Transforaminal epidural injections with steroids (TESI) are used increasingly for patients with sciatica. However there is much debate about their safety and effectiveness. It is important to identify patients that benefit most from TESI and only few trials have yet evaluated the effects in patients with acute sciatica.

**Methods:**

We describe a prospective, randomized controlled trial (RCT), with the aim to evaluate the hypothesis that TESI plus Levobupivacaine (TESI-plus) added to oral pain medication is more effective compared to pain medication alone or compared to transforaminal injection with a local anesthetic of short duration among patients with acute sciatica. We will recruit a total of 264 patients with sciatica (<8 weeks) caused by a herniated disc, from two clinical sites. Participants are randomly assigned one of three study groups: 1) oral pain medication (control group), 2) oral pain medication and TESI-plus (intervention group one), 3) oral pain medication and transforaminal epidural injection (TEI) with Levobupivaine and saline solution (intervention group two). Primary outcomes are functional status (Roland-Morris Disability Questionnaire), pain intensity for both leg and back, (100 mm visual analogous scale (VAS)), and global perceived recovery (GPR, reported on a 7-point Likert scale, dichotomized into ‘recovered’ and ‘not recovered’). The secondary outcomes are health-related quality of life (EQ5D-5 L) and patient satisfaction (7-point Likert scale). We will also collect information on healthcare utilization and costs, to perform an economic evaluation. All outcomes are measured at three and six weeks, three and six months after randomization. We defined a minimal clinically relevant difference between groups as a difference between both intervention groups and the control group of 20 points for pain (100-point VAS), four points for functional status (24-point RDQ) and a 20% difference on dichotomized GPR (recovered versus not recovered).

**Discussion:**

A clinically relevant outcome in favor of TESI-plus implies that future patients with acute sciatica should be recommended TESI-plus within the first few weeks rather than being treated with pain medication alone in order to relieve pain and improve their functioning. In case of a negative result (no relevant differences in outcome between the three study arms), pain medication will remain the mainstay of treatment in the acute stages of sciatica.

****Trial registration**:**

Dutch National trial register: NTR4457 (March, 6th, 2014)

## Background

Sciatica is characterized by neuropathic pain radiating from the lower back into the leg along following the sciatic nerve [1]. The principal source of the pain is nerve root impingement due to a mechanic compression: about 85% of cases of sciatica are caused by intervertebral disc herniation [2]. Patients may experience tingling or pricking in the dermatomal distribution of a nerve root, but sensory symptoms are usually minor [1]. Paresis is present in less than half of patients, for example foot drop due to weakness of the anterior tibial muscle (in case of L5 radiculopathy). The annual incidence of sciatica in The Netherlands is 9.4 cases per 1000 adults [3]. The economic effect of sciatica is major in terms of costs of hospital care and costs resulting from absenteeism from work and disability compared to any other disease category [4, 5].

During the first few weeks of symptoms treatment is focused on pain control by means of medication and mobilization by physical therapy. Disc surgery should only be proposed if symptoms persist after conservative treatment. There is no agreement on how much time (in terms of weeks) conservative therapy should be followed before surgery is advisable [6].

Epidural steroid injections are used increasingly as an alternative to pain medication in patients with sciatica, especially in acute patients with severe pain. In the United Kingdom, the number of epidural injections increased from 47 803 in 2000 to 70 967 in 2010 (increase of 49%) [7]. In a retrospective US cohort from 2000–2014 TESI against back pain increased 609% with an annual increase of 15% per 100 000 Medicare population [8].

There are three different techniques for epidural injection: caudal, interlaminar and tranforaminal. The original caudal approach was developed around 1900 by Sicard [9], and has largely been replaced by the other two methods. Most pain physicians in The Netherlands prefer a transforaminal approach, that is widely regarded as more effective than the interlaminar technique [10]. However, recent data show equivalence between the two [11, 12]. A wide variety of injections fluids is used, including local anesthetics (for example Procaine or Levobupivacaine) and glucocorticosteroids (including methylprednisolone and triamcinolone).

During recent years there has been discussion about the effectiveness and safety of epidural corticosteroids against sciatica. In their 2012 meta-analysis that included 23 trials, Pinto et al showed only a small but statistically significant, short-term (<3 months) effect for leg pain of epidural corticosteroids versus placebo (mean difference (MD), - 6.2 on a 100 point visual analogue scale (VAS) [95% CI, -9.4 to -3.0]) and disability (MD, - 3.1 on a 100 point Oswestry and (converted) Rolland Morris Disability scale [95% CI, -5.0 to - 1.2]) [13]. The pooled long-term effects (>12 months) were smaller and not significant. The level of evidence according to the GRADE approach was regarded as high [14]. Another meta-analysis of 30 trials concluded that epidural corticosteroid injections give greater immediate-term (<2 weeks) reduction in pain (MD -7.55 on a 100 point VAS [95% CI, −11.4 to −3.74]) and reduction in disability (standardized MD, −0.33 [95% CI, −0.56 to −0.09]) compared to placebo. The same analysis also showed a lower short-term (>2 weeks to < 3 months) surgery risk for patients treated with epidural corticosteroids (relative risk, 0.62 [95% CI, 0.41 to 0.92] [15].

In 2014, the American Food and Drug Administration (FDA) gave out a safety warning after several neurologic events had been reported in patients undergoing epidural corticosteroids, including some fatal events of spinal cord infarction and stroke [16, 17]. However, serious complications of injections below conus-level appear to be rare [16, 17]. Complications of epidural corticosteroids against sciatica are usually limited to nausea, headache, dizziness, vasovagal attacks and flushing of the face [20, 21].

It is important to select patients that benefit most from epidural corticosteroids while closely monitoring their safety [22, 23]. Given the fact that most patients with sciatica recover within three months [24, 25], and because biochemical markers of inflammation are elevated especially in patients with a short duration of symptoms [26, 27], there seems to be a window of opportunity with regards to the timing to treat patients with epidural corticosteroid injections (directed against inflammation) within the first weeks post onset of sciatica.

### Aim

The goal of study is to evaluate the effectiveness of TESI-plus and oral pain medication versus oral pain medication alone in improving pain, physical functioning and recovery among patients with sciatica within eight weeks after onset in outpatient clinics. Our hypothesis is that patients who are randomized to receive TESI-plus (intervention group one), will experience less pain and better functional status compared to patients randomized to receive pain medication alone (control group). A second hypothesis is that TESI-plus is more effective than transforaminal injection with Levobupivacaine and saline solution (intervention group two). Levobupivacaine is a local anesthetic with a short-lasting effect and is usually injected in a small volume. Its supposed effectiveness is minor. We are interested to see if the type of transforaminal epidural injection matters (using equal volumes).

## Methods

We followed the CONSORT statement when designing and reporting this study, a checklist invented to improve the quality of reports of RCTs [28].

### Study design

A multicenter, randomized controlled, prospective, single-blind trial will be performed, along with a full economic evaluation from a societal perspective. The subjects will be enrolled at two Dutch hospitals, the Zaans Medisch Centrum, Zaandam and Onze Lieve Vrouwe Gasthuis, Amsterdam. The two hospitals are located in a populated area of The Netherlands. The subjects will be allocated to one of three groups:

1. (Control) oral medication only;
2. (Intervention group one) oral medication and TESI-plus;
3. (Intervention group two): oral medication and TEI with Levobupivacaine and saline solution.

**Fig. 1:**
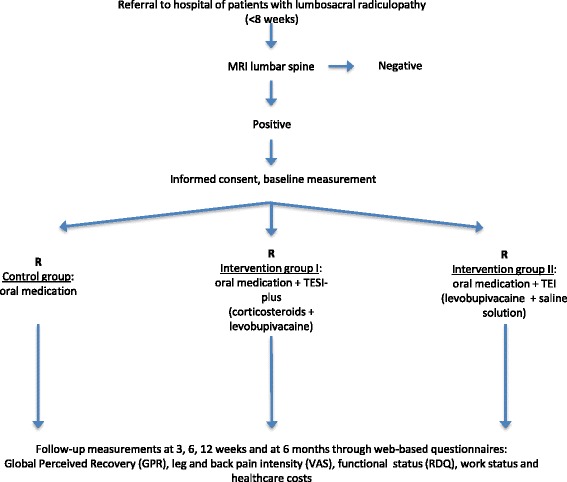
Trial design and patient flow

Follow-up will be six months. Figure 1 shows the study design and patient flow.

This trial will be carried out in accordance with the Declaration of Helsinki. The Boards of Directors of the two hospitals approved the execution of the study in their centers.

### Ethical approval

On August 20th 2015, the RCT was evaluated positively by the Medical research Ethics Committees United, Nieuwegein, The Netherlands (registration number NL 45805.100.15) and the protocol was registered at the Dutch Trial Register (number NTR 4457). All patients will give their (written) informed consent before participation in the trial.

### Study population

Patients eligible for this study have to have < 8 weeks of sciatic symptoms and will be seen by a neurologist of one of the two study centers upon referral by their general practitioners (GP). Additional inclusion criteria are: a) age between 18 and 75 years; b) a magnetic resonance imaging (MRI) confirmed disc herniation with nerve root impingement causing clinical symptoms; c) pain experienced on average over the last week rated on a numerical rating scale (NRS) (>4/10); d) good understanding of Dutch language; e) internet access in order to complete online questionnaires.

Exclusion criteria are: a) severe weakness of the legs (Medical Research Council ((MRC) score <3); b) spinal surgery < 1 year at the symptomatic lumbar level; c) lumbar spinal stenosis or spondylolisthesis as the cause of radicular pain diagnosed by MRI; d) pregnancy; e) severe comorbidity (e.g. cancer).

### Setting

Neurologists at the participating clinics will perform a complete physical and neurological examination. For specific description see ‘baseline measurement’. In case of a clinical suspicion of a herniated disc, MRI of the lumbar spine will be performed. If patients meet the eligibility criteria, they will get oral and written information about the trial. After being informed the patients will be asked to participate. Upon agreement, the informed consent form will be signed and patients will be randomized by one of the trial nurses. At the start baseline data will be registered. Transforaminal injections will be performed by an experienced anesthesiologist within two working days after randomization. After randomization the clinical and research settings will be separated: all patients will be followed by their own neurologist in the outpatient department and by the research nurse who is responsible for the (web based) questionnaires.

### Magnetic resonance imaging

Images will be made by a 1.5 T MRI scan, gradient strength 33 mT/m, slew rate 125 T/m/s (Siemens Magnetom Area, Siemens Medical Solutions, Erlangen, Germany) with a dedicated receive-only spine coil. All participants will be imaged with the same protocol. MR studies will start with a coronal plan scan (GRE; TR/TE = 4.20/2.38), followed by a sagittal T1-images (FSE; TR/TE/Etl = 660/9,90/3), a sagittal T2-images (FSE; TR/TE/Etl = 3530/96/17) and a transverse T2-images (FSE; TR/TE/Etl = 5380/91/15). The lumbar spine (T12-S1) will be studied on sagittal images, including imaging of the neural foraminae. Transverse images will be obtained from L3 to S2, with slices angulated parallel to the disci.

### Pain medication

Patients in the control and intervention groups use painkillers both over the counter and by prescription. Usually the GPs choose Paracetamol with or without non-steroidal anti-inflammatory drugs (NSAIDs) and, if necessary, opioids following the WHO-pain ladder [29]. In addition medication against neuropathic pain for example Pregabaline or Gabapentin, is often prescribed. All medication will be registered by online questionnaires. In case of kinesiophobia and/or a substantial inactive lifestyle patients are permitted to go to a physiotherapist. In summary there are no restrictions to pain medication or physiotherapy in all three study groups.

### Transforaminal epidural injections

The procedure is similar for both intervention groups. The study participant is brought to a fluoroscopy room and placed in a prone position on the procedure table. Fluoroscopy is used for localization of MRI confirmed disc herniation. Target identification and needle entry into the targeted space is done following internationally accepted procedures [30]. The skin is made sterile using chlorhexidine. The injections are given with 22 gauge 100 mm facet tipped needle (Pajunk RGN™). Right needle position is confirmed with the injection of 0.5-1.5 cc of Joversol 300 mg/ml contrast material (Optiray™ 300, Mallinckrodt). Once an image is obtained demonstrating contrast material spreading into the epidural space medial to a line connecting the ipsilateral lumbar vertebral pedicles, the injection is performed.

The study participants of intervention group one receive 1 ml of 5% Levobupivacaine followed by 1 ml of 40 mg/ml Methylprednisolone in an opaque syringe. The study participants of intervention group 2 receive 1 ml 5% Levobupivacaine followed by 1 ml NaCl 0.9%. The total volume of the two injections is the same (2 ml).

After the epidural injection the washout of the contrast fluid is demonstrated on an X-ray image. The image will be saved. Finally the needle is removed and the patient is brought to the recovery area.

### Baseline measurement & outcomes

We use the core outcome set for clinical trials in low back pain [31]. The questionnaires are web-based and will be completed at baseline, three weeks, six weeks, three and six months after randomization.

### Baseline measurement

The following potential prognostic factors for recovery at 6 months will be assessed at baseline: age, gender, education, profession, work and marital status, co-existing joint problems, presence of vascular risk factors (diabetes, hypertension) and all primary and secondary outcomes.) [32].

Neurological examination, performed by six different neurologists working in the outpatient departments of the participating hospitals, is standardized for all participants. Tests include physical examination of the leg muscles using the Medical Research Council (MRC) scale for muscle strength; sensory examination: tests for perception of light touch, pin prick, and vibration sense of the lower extremities; reflex examination: tests for reflexes in the patellar (L4) and ankle (S1); straight leg raising (or Lasègue’s sign): with the patient laying on the back, one leg is lifted upwards by flexing the hip while the knee remains extended. The test is positive if the patient experiences radicular pain when the leg is at an angle between 30 and 70°. A finger-floor distance of more than 25 cm, absence of knee or ankle tendon reflex, leg paresis and a positive straight leg raise test are an indication for a herniated disk with nerve compression on MRI [33]. The added value of a specified neurological examination is limited: most of the information revealed by physical testing will already follow from careful neurological history taking [34].

### Primary outcomes

The three primary outcomes are pain, physical functioning and global perceived recovery.

Pain intensity (average previous week) of both back and leg will be rated using a 100 mm VAS: 0 = no pain to 100 = worst imaginable pain [30]. The VAS is known as a valid and reliable measurement among back pain patients [35, 36].

GPR will be rated on a 7-point Likert scale that ranges from ‘completely recovered’ (-3) to ‘worse than ever’ (+3). The GPR will be dichotomized into success (categories ‘completely’ and ‘much recovered’) and non-success (categories ‘slightly recovered’, ‘no change’, ‘slightly worse’, ‘much worse’ and ‘worse than ever’). The GPR is a commonly questionnaire in back pain research [37].

Functional status will be rated using the Dutch version of the Roland-Morris Disability Questionnaire (RDQ) [38]. The RDQ counts 24 items for normal daily activities. Each question has a ‘yes’ or ‘no’ option. The RDQ ranges form 0–24 and is a valid and reliable tool that is commonly used back pain studies [38, 39].

A minimal clinically important difference is defined as an improvement of 20 points for pain (both leg and back) (100 point VAS), 4 points for functioning (24 point RDQ), and a 20% difference between groups for recovery (recovery vs. non recovery).

### Secondary outcomes

The Euroqol-5 dimensions- 5 levels (EQ-5D-5 L) will be used to determine quality of life [40]. The EQ-5D-5 L rates self-care, mobility, pain, psychic functioning (anxiety/depression), and usual activities on a 3-point scale (levels: no problems, moderate problems and severe problems). The EQ-5D-5 L is commonly used in cost-utility analyses and for that reason applied in the economic evaluation as well [39, 41].

Patient satisfaction will be assessed using a written 7-point NRS ranging from ‘not satisfied at all’ to ‘completely satisfied’. No gold standard is available for the measurement of patient satisfaction, but in spinal disorders a seven-point global question is recommended [31].

All measurements were registered using web-based questionnaire, which will be sent at baseline, and at three and six weeks, three and six months follow-up.

### Economic evaluation

The economic evaluation will focus on the comparison of intervention group 1 and the control group.

Intervention costs will be estimated using hospital accounting records. Health care utilization costs (i.e. primary care, secondary care, and the use of prescribed and over-the-counter medication), informal care and unpaid productivity will be collected using self-completed cost questionnaires at three weeks, six weeks, three and six months [42]. Work absenteeism, presenteeism, and productivity losses due to back- or leg pain will be measured by the Productivity and Disease Questionnaire (PRODISC). The PRODISC was validated in samples of patients and employees in The Netherlands [43]. It includes all relevant aspects of the link between health and productivity [44]. Absenteeism from paid work will be estimated by multiplying the total number of sickness absence days during follow-up by their associated costs, using the friction cost approach [45]. Guidelines from the handbook for economic evaluations in the Netherlands will be used [46].

Table 1 gives a schematic overview of data collection.

**Table 1:** Overview of the data collection

Outcome measures		Follow-up			
	Baseline	3 weeks	6 weeks	12 weeks	6 months
Baseline measurements					
Demographic data	X				
Prognostic factors	X				
Complaint history	X				
Physical examination	X				
MRI lumbar spine	X				
Primary outcomes					
Leg pain intensity (VAS)	X	X	X	X	X
Back pain intensity (VAS)	X	X	X	X	X
Global Perceived Effect (GPE)	X	X	X	X	X
Functional status (RDQ)	X	X	X	X	X
Secondary outcomes					
Work status	X	X	X	X	X
Quality of life (EQ-5D-5 L)	X	X	X	X	X
Drug use	X	X	X	X	X
Health care costs (journal)	X			X	X

### Adverse events and safety issues

All adverse events (AEs) during the study will be recorded on the case record form (CRF), whether or not caused by the study procedure. Registration includes: the event, onset and end date, severity, relation to the study and action taken. AEs considered related to the study will be judged by a medically qualified investigator and followed until resolution (or if the event is regarded stable). All AEs that result in withdrawal from the trial will be followed until there is satisfactory recovery. The investigator will judge whether an AE is severe enough to require the study participant’s removal. A study participant may withdraw from the trial if he or she experiences as an intolerable AE. If either side effect will happen, the study participant will get appropriate medical care until symptoms resolve or become stable. There will be an end of study assessment.

### Sample size

Sample sizes were calculated for the three primary outcomes pain, functional status and global perceived effect (for all: power 0.9; two-sided alpha 0.05). A number of 48 patients is needed in each arm to detect a difference of 20 points (VAS for both leg and back pain) between intervention group one and the control group between the intervention group one and two (SD 30). A 20 points difference is considered clinically relevant. A number of 22 patients in each arm is needed to detect a difference of 4 points on the RDQ (SD 4). A number of 79 patients in each arm is needed to detect a difference on the dichotomised GPE of 20%. We aim to include a total of 264 patients (*n* = 88 per arm) anticipating a 10% loss to follow up.

### Treatment allocation

Randomization will be performed by trial nurses using ALEA® software (NKI-AVL, The Netherlands). Alea® will generate a random schedule of blocks with maximum size of 6. A unique randomization number will be generated for each participant. An independent research nurse will allocate the participants to their group. Patients that belong to one of the intervention groups do not know the type of injection. Coding will not be broken during the trial.

### Blinding

This pragmatic trial is partially blinded. The patients do not know the type of injection received, but the anesthesiologist knows the different injection fluids. The neurologists that do the clinical follow-up of the study participants are blinded for the type of injection. The same applies to research nurses and the statistician. All patients will be assigned a unique number to ensure anonymity.

### Statistical analysis

Baseline characteristics will be compared of the main outcome measures, potential confounders (including: age, sex, and education) and prognostic factors.

The analysis will be performed according to the intention-to-treat method for all three comparisons (intervention group one versus intervention group two, intervention group one versus control group and intervention group two versus control group). All continuous variables will be analyzed using a maximum likelihood estimation for linear mixed models under ‘missing at random’ assumptions. In these analyses we will take into account the levels of patient, time of measurement and hospital, if necessary based on the likelihood ratio test. Regression coefficients and odds ratios with 95% confidence intervals (CI) for all follow up data, adjusted for baseline characteristics will be calculated, with a level of significance of *P* < 0.05.

All patients will be analyzed, regardless of the treatment received and violations from the study protocol. Secondly, the per-protocol analysis includes participants with all primary endpoint data available, and for who there haven been no major protocol violations. Protocol violations will be reviewed by the research team, blinded to allocation, and before locking the trial database. Data will be compared between complete and incomplete records to identify possible selective drop-out in the case of missing data.

### Economic evaluation

The economic evaluation will be done following an intention-to-treat approach and from a societal perspective.

A multivariate imputation (by chained equations) will be used to impute missing costs and effect data. Bootstrapping with 5000 replications will be used to estimate a 95% CI for differences in total costs between treatment groups.

Cost-effectiveness ratios will be calculated by dividing the difference in mean costs by the mean effect in pain intensity of the two treatment groups. Cost-utility will be expressed in costs per quality adjusted life year (QALY) and based on the EQ5D-5 L. Uncertainties with regard to cost-effectiveness and cost-utility ratios will be estimated using bootstrapping techniques and graphically shown in cost-effectiveness and utility planes. Acceptability curves for cost-effectiveness will also be made. Sensitivity analyses will be carried out for the most important cost drivers in order to assess the robustness.

## Discussion

Sciatica is considered to have three pathogenic components: a mechanic component that consists of impingement of the nerve root due to disc herniation; an inflammatory component that can be shown by elevated cytokines in serum and biopsies [26, 27]; a neuropathic component caused by neural damage.

We hypothesize that inflammation is predominant during the acute phase of sciatica and wanes after several months in correlation with clinical improvement in most patients. From this idea epidural corticosteroids that are administered locally at the site of the lesion are likely to be effective during the first weeks of an episode of sciatica. We could only find three previous RCTs that have addressed acute treatment of sciatica with epidural corticosteroids before [47–49]. Pooled data (unpublished) did not show significant relief from pain or disability in the corticosteroid group compared to placebo or care as usual. However, due to the low to moderate quality of evidence and the restricted number of studies included, a firm statement cannot be made based on these results.

A clinically relevant outcome in favor of TESI-plus implies that TESI-plus should be recommended for patients with acute sciatica within the first few weeks rather than being treated conservatively with pain medication alone in order to relieve pain and improve their functioning. In case of a negative result (no clinically relevant differences in outcome) pain medication remains the mainstay of treatment in the acute stages of sciatica.

Regardless of the outcome of our study surgery within the first 2–3 months is reserved for patients with severe pain irresponsive to medication or TESI and patients with neurological deficits (cauda syndrome or weakness).

Recently the safety of TESI has been debated in the literature [16–22]. Though the complication rate of TESI of the lumbar spine is known to be low - Manchikanti et al doing a thorough review could only identify several cases [18]- all subjects in our study will be closely monitored.

The trial started in February 2016. The results will be available at the end of 2017.

## Abbreviations

AE: Adversed effect
AR: Adversed reaction
CI: Confidence interval
CRF: Case record form
EQ-5D-5 L: Euroqol-5 dimensions- 5 levels
FDA: Food and drug administration
GP: General practitioner
GPR: Global perceived recovery
GRADE: Grades of recommendation, assessment, development and evaluation
MD: Mean difference
MRC: Medical research council
MRI: Magnetic resonance imaging
NRS: Numerical rating scale
NTR: Nederlands trial register
Prodisc: Productivity and disease questionnaire
Qaly: Quality adjusted life year
RCT: Randomized controlled trial
RDQ: Roland-morris disability questionnaire
TEI: Transforaminal epidural injection
TESI plus: Transforaminal epidural injections with corticosteroids and local anesthetic
TESI: Transforaminal epidural injections with steroids
VAS: Visual analogous scale
WHO: World Health Organization
